# Supplementary material for: The interactome of intact mitochondria by cross-linking mass spectrometry provides evidence for coexisting respiratory supercomplexes
Source: Mol Cell Proteomics. 2017 Dec 8;17(2):216–32. doi: 10.1074/mcp.RA117.000470 (PMC5795388; doi:10.1074/mcp.RA117.000470)
Supplement: Supplemental Data [file supp_RA117.000470_133922_0_supp_23978_fzffwf.zip › spectra_annotation/mito_spectra_annotation/411-1-20-1-59-2.pdf]

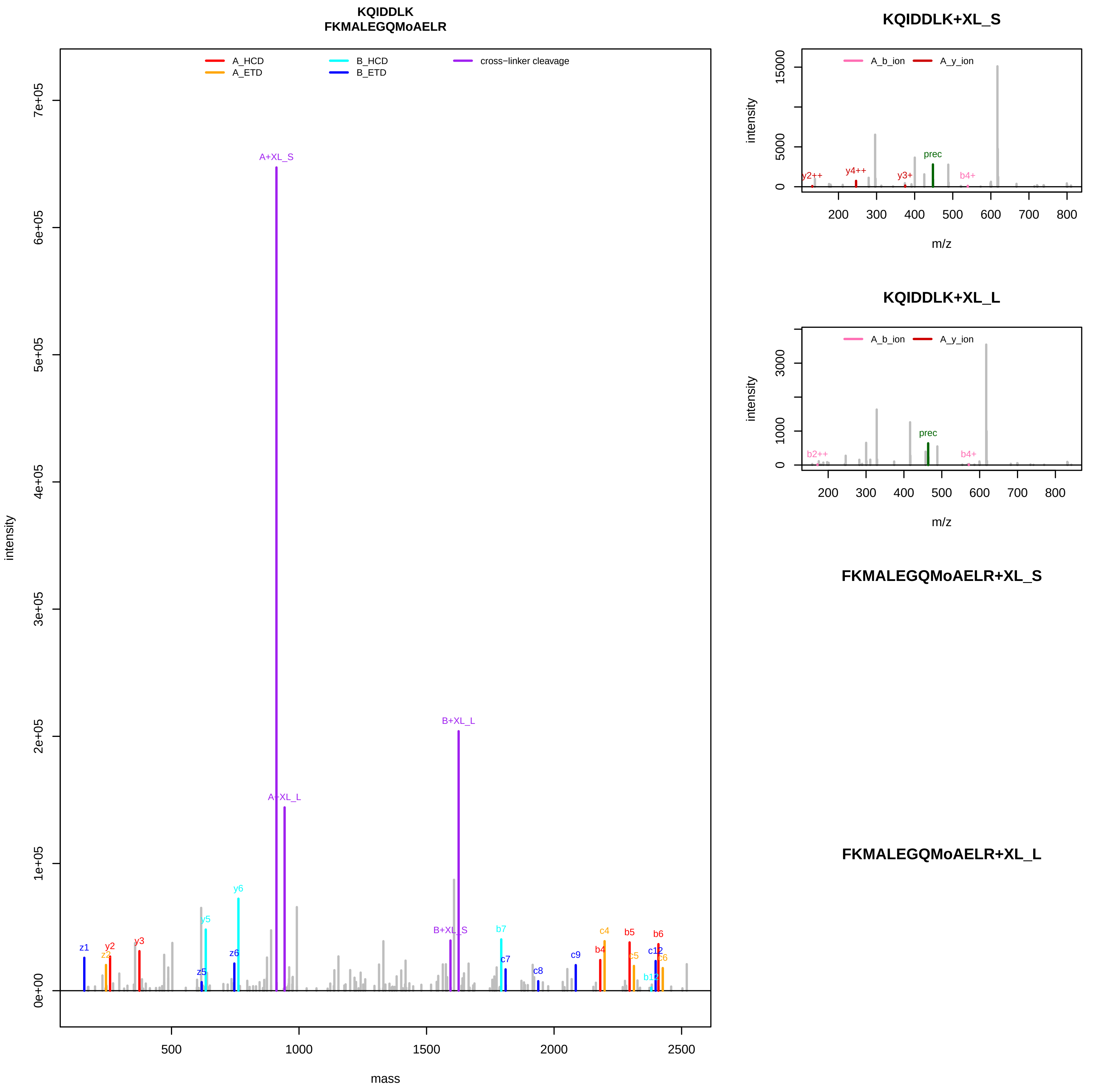
KQIDDLK
FKMALEGQMoAELR
A_HCD
A_ETD
B_HCD
B_ETD
cross−linker cleavage
A+XL_S
A+XL_L
B+XL_L
B+XL_S
y2
y3
y5
y6
z1
z2
z5
z6
b4
b5
b6
b7
b12
c4
c5
c6
c7
c8
c9
c12
intensity
mass
KQIDDLK+XL_S
A_b_ion
A_y_ion
y2++
y4++
y3+
prec
b4+
m/z
KQIDDLK+XL_L
b2++
FKMALEGQMoAELR+XL_S
FKMALEGQMoAELR+XL_L
